# The Role of Nutrition and Body Composition on Metabolism

**DOI:** 10.3390/nu16101457

**Published:** 2024-05-12

**Authors:** Yuyang Wang, Botian Chen, Defu Ma

**Affiliations:** Department of Social Medicine and Health Education, School of Public Health, Peking University Health Science Center, Beijing 100191, China; wyuyang2018@163.com (Y.W.); cbt970812@163.com (B.C.)

## 1. Introduction

Metabolism encompasses the entire array of chemical reactions continuously occurring within the body that sustain life and maintain normal physiological functions. These reactions involve both the breakdown of nutrients derived from food and the synthesis and repair of the body tissues [1,2]. Nutrition refers to the intake and utilization of nutrients from the diet to support life processes. Metabolism represents a complex, coordinated process that transforms these nutrients into usable substrates. Significantly, the delicate balance between nutrition and metabolism can be substantially altered by nutritional disorders ranging from undernutrition to overnutrition [3].

Within the realm of human health and disease, the interplay between nutrition, body composition, and metabolic processes is of fundamental importance. These three factors collectively shape an individual’s physiological state, affecting energy balance, weight management, and the risk of chronic diseases [4,5,6]. Nutrition, as an indispensable element for maintaining life activities, directly influences the body’s physiological functions, and the intake and utilization of nutrients are closely linked to metabolic processes. Body composition not only reflects an individual’s nutritional status but also serves as a primary site for metabolic activity, particularly muscle tissue and body fat distribution [7,8]. Their impact on energy expenditure and storage mechanisms further elucidates the complex mechanisms by which body composition regulates metabolism.

This Special Issue includes ten original research articles and one review, which collectively explore the multifaceted interactions between nutrition, body composition, and metabolism. These new findings offer diverse insights into understanding the homeostatic processes within the body.

## 2. A Summary of Published Articles

Nutrients are essential in the metabolic processes within the human body. Fu and colleagues (contribution 1) explored the association between Vitamin B levels and obesity among middle-aged and older Chinese adults. Their study revealed that the levels of Vitamin B (B_1_, B_2_, B_6_, and B_9_) are negatively correlated with obesity, as defined by body fat percentage (BF%), visceral fat area (VFA), or waist circumference (WC). This main conclusion was also observed among female participants and older adults. Echoing Fu’s findings, Dang and colleagues (contribution 2) demonstrated that the predictive power of metabolic syndrome (MetS) in Vietnamese adults varies with the choice of body composition indicators. Therefore, Dang and colleagues identified the optimal anthropometric indices and cutoff values for predicting metabolic syndrome in Vietnamese adults. Karpińska and colleagues (contribution 3) evaluated the association between the body composition of young women and their intake of particular nutrients. After surveying 478 participants, their findings revealed that 37% of those with a normal body mass index (BMI) had high levels of body fat. Furthermore, the proportion of energy derived from plant-based proteins showed an inverse association with both the percentage of body fat (PBF) and BMI.

Besides the relationship between nutrients and body composition, the impact of various dietary habits on identity components or metabolism is one of the highlights of this Special Issue. Kwiatkowska and colleagues (contribution 4) investigated the potential relationships between dietary habits and body composition in individuals across different dietary patterns. Their research demonstrated that the Vegan group had the lowest mean values of body fat mass (BFM), PBF, and visceral adipose tissue (VAT) in comparison to other groups. Furthermore, Bučan Nenadić and colleagues (contribution 5) discovered that body composition metrics, including Phase Angle (PhA), can serve as indicators of metabolic syndrome (MetS) and reflect adherence to Mediterranean diet (MeDi) guidelines. “This insight can guide patients in improving their body composition and choosing healthier dietary patterns. Meanwhile, Zhao (contribution 6) reviewed the outcomes of ketogenic diets in cancer patients from prior controlled trials, verifying that ketogenic diets are a viable and safe approach for cancer patients to lower body weight and fat mass.

Multiple sclerosis (MS) is an autoimmune disease characterized by complex interactions between genetic susceptibility and environmental factors. Matusik and colleagues (contribution 7) evaluated the nutritional status of MS patients and explored its correlation with clinical state, duration of MS, and history of previous glucocorticoid therapy. The study results reveal a strong association between greater disability severity and reduced fat-free mass, as well as increased fat mass, especially concerning abdominal fat distribution in patients with MS. Guan and colleagues (contribution 8) assessed the relationship between basal metabolic rate (BMR) and body water distribution with muscle mass, and investigated dietary risk factors contributing to muscle wasting, sarcopenia, and sarcopenic obesity. Their findings suggest that in elderly individuals, elevated BMR and BMR/body surface area (BSA) act as protective factors against sarcopenic obesity and sarcopenia. Furthermore, a moderate intake of dietary carbohydrates may enhance the skeletal muscle index (SMI) in the elderly by influencing BMR and body water distribution.

Dou and colleagues examined the variability of human milk oligosaccharides (HMOs) in mothers with gestational diabetes mellitus (GDM). They assessed HMO concentration dynamics during lactation in exclusively breastfeeding mothers with GDM and compared them with healthy controls. The study revealed that the majority of HMOs exhibited a notable temporal decrease in concentration throughout the lactation period (contribution 9).

This Special Issue also features a report by Reynolds and Ives (contribution 10) on animal experiments investigating the role of PAR2 in age-related obesity. The study findings indicate that genetic deletion of PAR2 did not mitigate the rise in adiposity typically seen with aging. Contrarily, PAR2KO-AG mice exhibited increased body mass and fat mass relative to age-matched control C57BL6 mice. Guerrier and colleagues (contribution 11) provided a narrative review outlining the role of mitochondria in human white adipose tissue (AT). Despite relatively low mitochondrial density in white AT, their review underscores its critical role in energy metabolism. Moreover, the review synthesizes evidence from the current literature pointing to a likely link between mitochondrial function in AT and obesity.

## 3. Conclusions

This Special Issue explores the interactions between nutrition, body composition, and metabolism from different perspectives. Researchers have focused on the nutritional and mechanistic aspects related to obesity, diabetes, hypertension, metabolic syndrome, and exercise physiology, offering numerous discoveries and perspectives. As the understanding of the effects and mechanisms of nutrition and body composition on metabolism continues to evolve, there is growing scientific evidence underscoring the importance of maintaining healthy dietary habits, balanced nutritional intake, appropriate body composition parameters, and body homeostasis.
